# Feasibility of radiation dose reduction with iterative reconstruction in abdominopelvic CT for patients with inappropriate arm positioning

**DOI:** 10.1371/journal.pone.0209754

**Published:** 2018-12-31

**Authors:** Nieun Seo, Yong Eun Chung, Chansik An, Jin-Young Choi, Mi-Suk Park, Myeong-Jin Kim

**Affiliations:** Department of Radiology, Yonsei University Health System, Yonsei University College of Medicine, Seodaemun-gu, Seoul, Korea; BRF, UNITED STATES

## Abstract

**Background:**

The arms-down position increases computed tomography (CT) radiation dose. Iterative reconstruction (IR) could enhance image quality without increasing radiation dose in patients with arms-down position.

**Aim:**

To investigate the feasibility of reduced-dose CT with IR for patients with inappropriate arm positioning

**Methods:**

Twenty patients who underwent two-phase abdominopelvic CT including standard-dose and reduced-dose CT (performed with 80% of the radiation dose of the standard protocol) with their arms positioned in the abdominal area were included in this study. Reduced-dose CT images were reconstructed using filtered back projection (FBP), hybrid IR, and iterative model reconstruction (IMR). These images were compared with standard-dose CT images reconstructed with FBP. Objective image noise in the liver and subcutaneous fat was measured by standard deviation for the quantitative analysis. Then, two radiologists qualitatively assessed beam hardening artifacts, artificial texture, noise, sharpness, and overall image quality in consensus.

**Results:**

Reduced-dose CT with all IR levels had lower objective image noise compared to standard-dose CT with FBP reconstruction (P < 0.05). Quantitatively measured beam hardening artifacts were similar in reduced-dose CT with iDose levels 5–6 and fewer with IMR compared to standard-dose CT. In the qualitative analysis, beam hardening artifacts and noise decreased as the IR levels increased. However, artificial texture was significantly aggravated with iDose 5–6 and IMR, and overall image quality significantly worsened with IMR.

**Conclusions:**

IR algorithms can reduce beam hardening artifacts in a reduced-dose CT setting in patients with arms-down position, and an intermediate level of hybrid IR allows radiologists to obtain the best image quality. Because the retrospective and single-center nature of our study limited the number of patients, multicenter prospective clinical studies are required to validate our results.

## Introduction

Abdominopelvic computed tomography (CT) is usually performed with arms positioned above shoulders because beam hardening artifacts and quantum mottle/photon starvation caused by arms in the scan range negatively affect image quality and increase radiation exposure. However, some patients cannot raise their arms because of medical conditions such as trauma, drowsy mental status, or neurologic disease. The automatic exposure control (AEC) technology widely used in multi-detector CT systems automatically adjusts the tube current to the patient’s attenuation, guaranteeing good image quality at the lowest possible radiation dose independent of patient body volume [1–3]. Thus, AEC in CT scanners was expected to enable the scanning of patients with their arms positioned along the abdominal area while preserving good image quality. However, as AEC does not appropriately compensate for large body volume arising from arm-down positions, the degree of artifacts and radiation dose increases with AEC [4,5].

Several alternatives have been suggested to attain the best image quality in traumatized patients with incorrect arm positioning [5,6]. These methods have focused on modifying the arm position. For example, in patients who cannot elevate both arms, better image quality can be achieved by raising at least one arm [5]. In cases in which shoulder or arm injuries preclude arm raising, the positioning of both arms in front of the upper abdomen is better than positioning both arms alongside the torso [5]. However, arms should be manipulated carefully because unexpected plexus injuries may occur, although the incidence of such injuries is low [7,8].

Iterative reconstruction (IR) algorithms are reconstruction methods extensively used to preserve image quality without increasing radiation doses during CT scans and have been proven to improve image quality compared to filtered back projection (FBP) reconstruction at the same radiation dose [9–13]. Considering that IR reduces image noise and artifacts compared to FBP reconstruction, we hypothesized that IR would help radiologists obtain images of acceptable quality in patients unable to raise their arms without a significant increase in radiation exposure. To our knowledge, there has been only one study that compared artifacts in CT images between FBP, full IR and hybrid IR without arm elevation [14]. However, the prior study did not focus on reduced-dose CT with IR in patients with arms-down position. Therefore, the purposes of this study were to investigate the feasibility of performing reduced-dose abdominopelvic CT with IR in patients with inappropriate arm positioning and to assess the optimal IR protocol for clinical use.

## Methods and materials

### Patients

This retrospective study was approved by institutional review board in the Severance Hospital, and informed consent was waived due to retrospective study design. In our hospital, CT technicians asked patients if they could raise their arms above their shoulders before CT scanning. In cases in which patients could not raise their arms or could not answer because of a drowsy mental status, their arms were positioned alongside the torso for CT scanning. From July 2015, a two-phase abdominopelvic CT protocol for patients with inappropriate arm positioning consisted of reduced-dose scanning in the late arterial phase (LAP) and standard-dose scanning in the hepatic venous phase (HVP), to reduce radiation exposure during CT. Based on a previous phantom study result (S1 File), the radiation dose of the LAP was set to 80% of that of the standard protocol by decreasing the Dose Right Index (DRI). DRI is a discrete parameter designed to give consistent image quality for every patient size. By setting DRI, the default mAs is determined according to age group and patient size. After determining the default mAs using DRI, AEC is also applied. Using DRI, we attempted to adjust the radiation exposure of the LAP so that the patients would be exposed to similar amounts of radiation with arms positioned alongside the torso and arms located outside the scanning field. From July 2015 to February 2016, 20 consecutive patients (male:female ratio, 11:9; mean age ± SD, 66.7 ± 12.8 years) who underwent two-phase abdominopelvic CT scans with unavailable standard arms-up position were included in this study. The reasons for CT examinations in these patients were as follows: follow-up of underlying malignancy (n = 13; 4 cases of stomach cancer, 4 cases of colon cancer, 3 cases of lymphoma, and 2 cases of lung cancer), gastrointestinal bleeding (n = 2), fever (n = 3), and abdominal pain (n = 2). The mean body weight of these 20 patients was 57.4 ± 12.3 kg (range, 35–81), and the mean body mass index (BMI) was 21.8 ± 4.6 kg/m^2^ (range, 15.6–31.6). Among these patients, 16 patients could not raise both arms and four patients could not raise one arm.

### CT acquisition and radiation dose measurements

CT scans were performed with a 128 slice CT with double z-sampling (Brilliance iCT, Philips Healthcare, Cleveland, OH, USA). CT was performed after intravenous injection of 2.0 mL/kg (up to a maximum 150 mL when patients weighed more than 75 kg) of iodinated contrast media (Omnipaque 300, Iohexol, GE Healthcare, Cork, Ireland) followed by a bolus injection of 20 mL of saline chaser. CT scans in the LAP and HVP were obtained 18 s and 55 s after the attenuation increased 100 HU compared to the baseline in the abdominal aorta. Identical CT parameters were used for the post-contrast scans in the LAP and HVP except for the DRI (radiation dose) and were as follows: applied automatic exposure control; reference kV, 120 kV; pitch, 0.899; rotation time, 0.5 s; beam collimation, 128× 0.625 mm, and section thickness, 3 mm. The DRI was set to 15 for the HVP and 13 for the LAP.

The CT dose index volume (CTDIvol) and dose length product (DLP) in the LAP and HVP were recorded. Then, the size-specific dose estimate (SSDE) was estimated from CTDIvol by using a conversion factor based on the water equivalent diameter, which was provided by the American Association of Physicists in Medicine [15].

### Image reconstruction

Two IR methods, iDose and iterative model reconstruction (IMR), were used in this study. The iDose is a hybrid IR algorithm, and its levels (1 to 6 or 7 depending on the scan protocol) define the degree of noise removal. An increase in the iDose level indicates an increase in the strength of noise removal, with an approximately 11%–55% increase being reported relative to the corresponding FBP reconstruction [16]. IMR is an advanced model- and knowledge-based IR algorithm [17]. Image noise is reduced according to three IMR levels (1–3), and higher levels indicate larger reductions in noise.

Standard-dose CT scans (for the HVP) were reconstructed with FBP whereas reduced-dose CT scans (for the LAP) were reconstructed using FBP and the two previously mentioned IR algorithms in the console. Therefore, nine imaging sets were generated for IR: iDose levels 1 to 6, and IMR levels 1 to 3. A total of 11 datasets, i.e., 1 FBP set each for the standard-dose and reduced-dose CT scans, and 9 IR sets for the reduced-dose CT scans, were analyzed for each patient. The reconstructed slice thickness was 3 mm without overlap, and was the same for all reconstruction image sets.

### Quantitative image analysis

All quantitative measurements were made using software Image-J version 1.51 (http://imagej.nih.gov/ij). For all measurements, the size, shape, and location of the regions of interest (ROIs) were the same between the standard-dose CT images and the reconstructed reduced-dose CT image by using the copy-and-paste function of the software. One radiologist (with 4 years of experience in abdominal CT) measured objective image noise, which was defined as the standard deviation (SD) of the mean CT number within a ROI. Objective image noises were measured in subcutaneous tissue and liver. First, a circular ROI (21.0–582.2 mm^2^) was placed in the subcutaneous fat tissue of the abdominal wall to include an area with beam hardening artifacts generated by the arm(s) in the abdominal area. Second, three circular ROIs (72.9–785.7 mm^2^) were positioned in the severely affected areas of the liver with beam hardening artifacts, and the mean value of these ROIs was used in the analysis. The areas of blood vessels, bile ducts, and focal hepatic lesions were avoided in the ROI measurements. In addition, the difference between the maximum HU and minimum HU within each ROI, which represents the bright and dark streaks of the beam hardening artifacts, respectively, was calculated to assess changes in the beam hardening artifacts after IR was applied. Finally, the corrected CT number (HU), defined as [(maximum HU–minimum HU)/mean HU], was used in the analysis to adjust for the effect of different attenuation (or enhancement) of the liver between the LAP and HVP.

### Qualitative image analysis

Two board-certified radiologists with 3 and 9 years of experience in abdominal CT reviewed the CT images in consensus. Two imaging sets, a standard-dose CT set and each reconstructed image set of reduced-dose CT, were displayed side by side for comparative analysis. The window width and level was fixed at 350 and 50, respectively throughout the reconstructed image set. As clinical abdominal CT images from this CT scanner routinely include images reconstructed with IMR1 and iDose3, the raters were experienced with IMR1 and iDose3. Beam hardening artifacts, subjective image noise, artificial texture, margin sharpness, and overall image quality were graded using a five-point scale (S2 File). All reduced-dose CT images were scored compared to the standard-dose CT images. For beam hardening artifacts, artificial texture and image noise, scores were graded as follows: 1, much more beam hardening artifacts (artificial texture or image noise) than the standard-dose CT; 2, slightly more than the standard-dose CT; 3: similar to the standard-dose CT; 4, slightly less than the standard-dose CT; and 5: much less than the standard-dose CT. Margin sharpness and overall image quality were graded as follows: 1, much poorer than the standard-dose CT; 2, slightly poorer than the standard-dose CT; 3, similar to the standard-dose CT; 4, slightly better than the standard-dose CT; and 5, much better than the standard-dose CT.

### Statistical analysis

The differences in quantitative image noise between the standard-dose CT images and reconstructed image sets of the reduced-dose CT were compared using the paired *t*-test. The Wilcoxon signed rank test was used to compare the qualitative scores of beam hardening artifacts, artificial texture, subjective image noise, margin sharpness, and overall quality of the standard-dose CT and reduced-dose CT images (S3 File). Statistical analyses were conducted using software SPSS version 23.0 (IBM Corp., New York, NY). *P* values smaller than 0.05 were considered statistically significant.

## Results

### Radiation dose measurements

The mean CTDIvol of the standard-dose CT was 7.0 ± 1.8 mGy (range, 4.9–11.9) and the DLP was 439.9 ± 127.0 mGy·cm (276.8–796.3). The mean SSDE of the standard-dose CT was 9.8 ± 1.4 mGy. The mean CTDIvol of the reduced-dose CT was 5.7 ± 1.8 mGy (3.9–11.1) and the DLP was 358.8 ± 124.0 mGy·cm (220.3–742.8). The mean SSDE of the reduced-dose CT was 8.0 ± 1.4 mGy. The mean SSDE of the reduced-dose CT was 18.4% less than that of the standard-dose CT.

### Quantitative analysis

The results of quantitative measurement and analysis are summarized in Table 1. The objective image noise of the subcutaneous fat area with beam hardening artifacts in the reduced-dose CT with FBP was significantly higher than in the standard dose-CT with FBP (*P* < 0.001). The objective image noise of subcutaneous fat decreased as the iDose and IMR level increased. The objective image noises of subcutaneous fat in the reduced-dose CT with iDose levels between 4 and 6 and all IMR levels were significantly lower than those in the standard dose-CT with FBP (*P* < 0.05). In the liver with beam hardening artifacts, the objective image noises in the reduced-dose CT were significantly lower with all IR levels than that in the standard-dose CT with FBP (*P* < 0.05). The corrected CT numbers [(max–min)/mean value] for all IMR levels in the reduced-dose CT were significantly lower than those in the standard-dose CT with FBP (*P* < 0.05).

**Table 1:** Objective image noise (OIN) of standard-dose CT and reduced-dose CT.

	Standard- dose	Reduced-dose									
	FBP	FBP	iDose 1	iDose 2	iDose 3	iDose 4	iDose 5	iDose 6	IMR 1	IMR 2	IMR 3
OIN of Subcutaneous fat	19.7 ± 5.6	24.9 ± 8.6	20.9 ± 8.4	19.4 ± 6.7	18.5 ± 6.8	16.9 ± 5.8	16.0 ± 7.7	15.1 ± 7.4	11.3 ± 5.5	9.4 ± 4.3	8.3 ± 3.7
*P*-value		< 0.001	0.381	0.789	0.258	0.007	0.007	0.003	< 0.001	< 0.001	< 0.001
OIN of Liver	23.9 ± 7.1	25.1 ± 5.3	21.1 ± 3.8	20.1 ± 3.7	18.9 ± 3.6	17.9 ± 3.6	16.6 ± 3.7	15.9 ± 4.2	11.7 ± 3.8	10.8 ± 4.0	10.1 ± 4.1
*P*-value		0.225	0.009	0.001	< 0.001	< 0.001	< 0.001	< 0.001	< 0.001	< 0.001	< 0.001
(max-min)/mean CT density (HU)	1.5 ± 0.7	2.5 ± 1.1	2.1 ± 1.0	2.0 ± 1.0	1.9 ± 0.9	1.8 ± 0.9	1.7 ± 0.8	1.6 ± 0.8	1.1 ± 0.6	0.9 ± 0.6	0.8 ± 0.6
*P*-value		< 0.001	< 0.001	< 0.001	0.008	0.038	0.234	0.710	0.001	< 0.001	< 0.001

Data are mean ± standard deviation.

*P*-values are for differences between standard-dose FBP and each reconstruction of reduced-dose CT.

### Qualitative analysis

**Fig 1:**
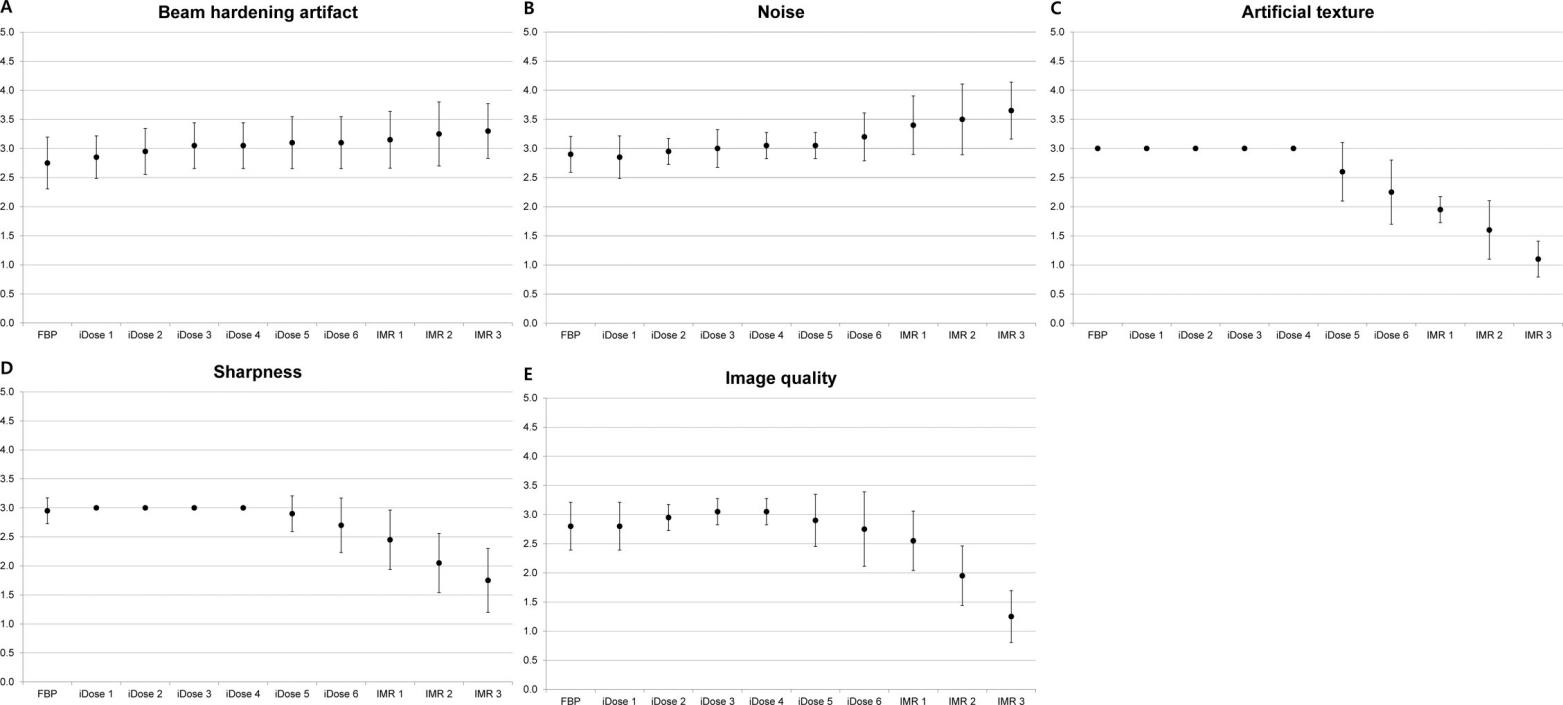
Graphs illustrating qualitative scores of each reconstruction set of the reduced-dose CT. All reduced-dose CT images were scored compared to the standard-dose CT images (e.g., 3: similar to the standard-dose CT). Higher score indicates a better image quality. (A) Beam hardening artifacts and (B) subjective image noise decreased as iDose and IMR levels increased. However, iDose levels of 5 and 6, and all IMR levels negatively affect (C) artificial texture and (D) margin sharpness. (E) Overall image quality was highest with iDose levels of 3 and 4.

**Fig 2:**
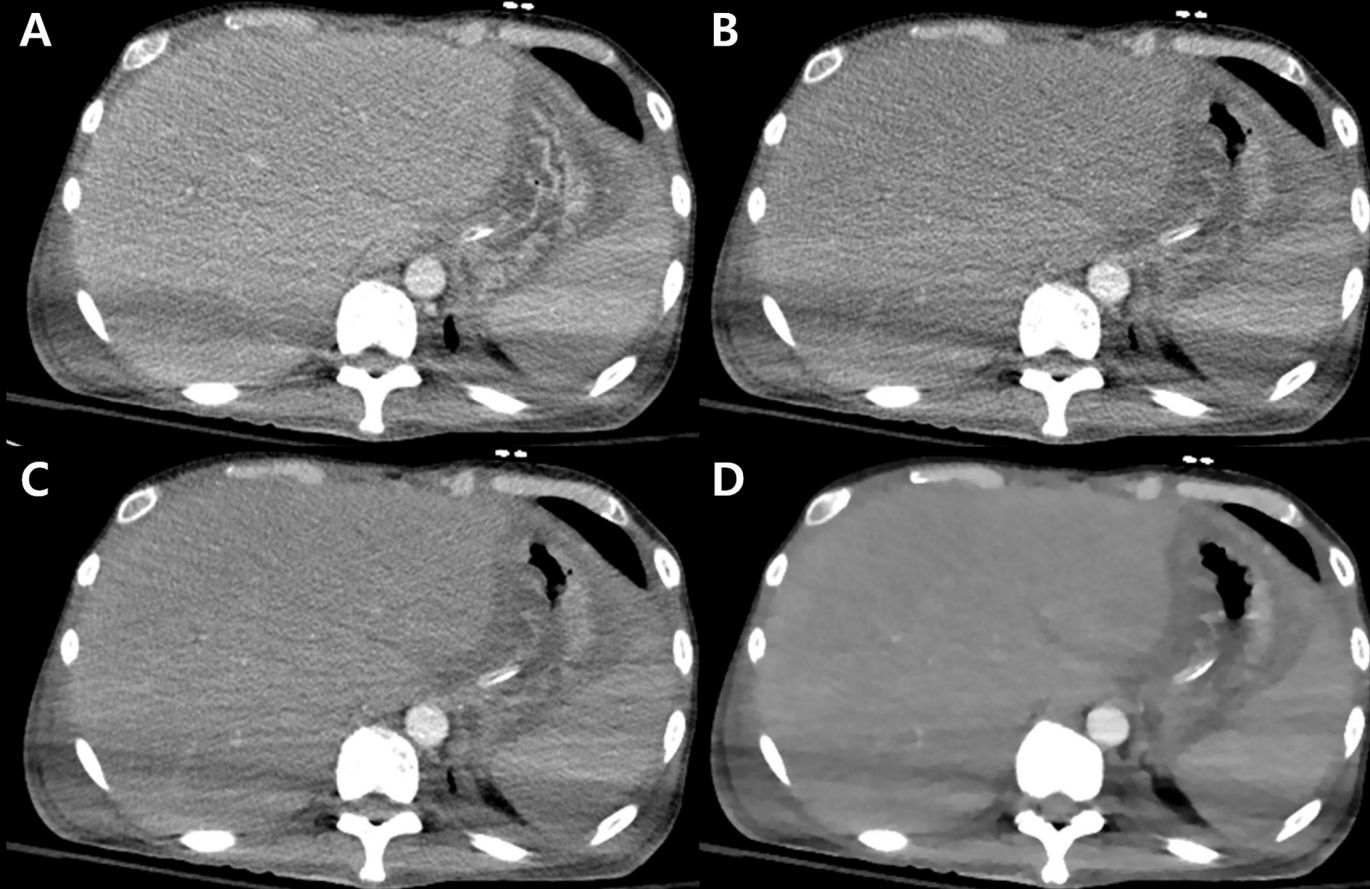
**Multidetector CT images (A: standard-dose CT image; B-D: reduced-dose CT images) of a 45-year-old male patient with his two arms positioned on his sides.** The section thickness was 3 mm. The window width and level was fixed at 350 and 50, respectively throughout the reconstructed image set. (A) The standard-dose CT image with filtered back projection (FBP) reconstruction shows the presence of beam hardening artifacts associated with the arms-down position. (B) The reduced-dose CT image with FBP reconstruction shows more prominent hypodense and hyperdense artifacts in the liver and spleen compared with (A). (C) A reduced-dose CT image with hybrid iterative reconstruction (iDose level 4) had similar or slightly less beam hardening artifacts than (A) while image quality was preserved. (D) A reduced-dose CT image with iterative model reconstruction (IMR level 3) shows less beam hardening artifacts than (A-C). However, IMR negatively affected the overall image quality due to prominent artificial texture and poor margin sharpness.

Fig 1 and Table 2 shows the results of qualitative analysis. The beam hardening artifacts in the reduced-dose scan with FBP reconstruction were significantly greater than those in the standard-dose scan with FBP reconstruction (*P* = 0.025). When iDose or IMR was applied, the degree of beam hardening artifacts decreased as the IR level increased. Similarly, the subjective image noise decreased as the iDose and IMR levels increased. However, artificial texture and poor image sharpness increased as the iDose and IMR levels increased (Fig 2). The reviewers observed a strong artificial texture and degraded sharpness in the reduced-dose scans with iDose levels between 5 and 6, and all IMR levels (*P* < 0.05). The overall image quality was highest with iDose levels of 3 and 4. IMR of all levels significantly decreased image quality compared to standard-dose CT scans with FBP reconstruction (*P* < 0.05).

**Table 2:** Subjective image quality scores of reduced-dose CT compared with standard-dose CT*.

	FBP	iDose 1	iDose 2	iDose 3	iDose 4	iDose 5	iDose 6	IMR 1	IMR 2	IMR 3
Beam hardening	2.8 ± 0.4	2.9 ± 0.4	3.0 ± 0.4	3.1 ± 0.4	3.1 ± 0.4	3.1 ± 0.4	3.1 ± 0.4	3.2 ± 0.5	3.3 ± 0.6	3.3 ± 0.5
*P*-value	0.025	0.083	0.564	0.564	0.564	0.317	0.317	0.180	0.059	0.014
Artificial texture	3.0 ± 0.0	3.0 ± 0.0	3.0 ± 0.0	3.0 ± 0.0	3.0 ± 0.0	2.6 ± 0.5	2.3 ± 0.6	2 ± 0.2	1.6 ± 0.5	1.1 ± 0.3
*P*-value	> 0.999	> 0.999	> 0.999	> 0.999	> 0.999	0.005	< 0.001	< 0.001	< 0.001	< 0.001
Noise	2.9 ± 0.3	2.9 ± 0.4	3.0 ± 0.2	3.0 ± 0.3	3.1 ± 0.2	3.1 ± 0.2	3.2 ± 0.4	3.4 ± 0.5	3.5 ± 0.6	3.7 ± 0.5
*P*-value	0.15	0.083	0.317	> 0.999	0.317	0.317	0.046	0.005	0.004	< 0.001
Sharpness	3.0 ± 0.2	3.0 ± 0.0	3.0 ± 0.0	3.0 ± 0.0	3.0 ± 0.0	2.9 ± 0.3	2.7 ± 0.5	2.5 ± 0.5	2.1 ± 0.5	1.8 ± 0.6
*P*-value	0.317	> 0.999	> 0.999	> 0.999	> 0.999	0.157	0.014	0.001	< 0.001	< 0.001
Image quality	2.8 ± 0.4	2.8 ± 0.4	3.0 ± 0.2	3.1 ± 0.2	3.1 ± 0.3	2.9 ± 0.4	2.8 ± 0.6	2.6 ± 0.5	2.0 ± 0.5	1.3 ± 0.4
*P*-value	0.046	0.046	0.317	0.317	0.317	0.317	0.096	0.003	< 0.001	< 0.001

Data are mean ± standard deviation.

*All reduced-dose CT images were scored compared with the standard-dose CT images (e.g., 3: similar to the standard-dose CT)

*P*-values are for differences between standard-dose FBP and each reconstruction of reduced-dose CT.

## Discussion

Our results showed that the image quality of reduced-dose scans with moderate levels of IR was similar to that of standard-dose scans with FBP reconstruction in patients who could not raise their arms during CT scanning. This finding indicates that additional radiation exposure due to the arms-down position can be avoided while maintaining image quality using IR. This study was the first to investigate the feasibility of reduced-dose CT with IR in patients with inappropriate arm positioning. Objective and subjective image noise, including beam hardening artifacts, decreased as the IR levels increased. However, as artificial texture and poor image sharpness deteriorated image quality at iDose levels between 5 and 6 and all IMR levels, the overall image quality was highest at iDose levels between 3 and 4.

CT scans performed with arms along the body are not rare, especially in trauma patients or critically ill patients. Skeletal surveys using CT are also performed on multiple myeloma patients with the arms-down position on a routine basis [18]. In these cases, radiation exposure is known to increase compared to the standard arms-up position [4,6,19]. A previous study reported the corrected effective dose to be 18% higher in patients with one arm down and 45% higher in patients with two arms down [4]. We sought to keep similar radiation exposure to the standard arms-up position while maintaining image quality using IR in these patients. As we hypothesized, the quality of CT images obtained with IR at a lower radiation dose (18.4%) was similar to that of FBP images obtained with the standard radiation dose. The objective and subjective image noise of reduced-dose CT scans was even lower than the image noise of standard-dose CT scans when higher levels of iDose or IMR were applied to the reduced-dose CT scans.

One recent study using GE-CT and Toshiba-CT reported that full IR allowed more efficient streak artifact reduction compared with FBP and hybrid IR in abdominal CT without arm elevation [14]. However, the previous study only evaluated unenhanced CT images, and did not investigate the feasibility of reduced-dose CT with IR in patients without arm elevation. In our study, CT images with IMR also showed significant reduction of beam hardening artifacts. However, the severe blotch appearance of IMR significantly decreased the overall quality of the CT images. Other several studies on IR have evaluated beam hardening artifacts due to metallic materials [20–22]. A phantom study on prosthetic heart valve CT imaging demonstrated that IR significantly decreased hypodense and hyperdense artifact volumes by 53% and 20%, respectively, for iDose, and by 67% and 23%, respectively, for IMR, compared with FBP [20]. However, subjective image quality could not be assessed because this study was a phantom study. Although beam hardening artifacts due to metallic materials and those due to non-metallic materials might be somewhat different because metallic materials produce much higher attenuation, resulting in severe photon starvation and beam hardening, our study indicated that IR could be used to reduce beam hardening artifacts generated by the positioning of arms next to the torso.

The two IR algorithms investigated in this study, iDose and IMR, have distinct mechanisms for noise reduction. iDose is a hybrid IR algorithm that works in the projection and image domains [23]. IMR is a model-based algorithm that is fully iterative with forward and backward reconstructions [24–26]. The most significant difference between iDose and IMR is that IMR decreases quantum noise and non-random noise intrinsic to the geometry and optics of the imaging system [20]. Therefore, IMR was expected to have a greater impact on the reduction of beam hardening artifacts, and this was demonstrated in this study. Despite significant improvements in objective and subjective image noise and beam hardening artifacts with IMR, the overall subjective image quality was significantly poorer with IMR than with standard-dose FBP images. It seems that objective image quality does not exactly correlate with subjective rating, which could also be dependent on the reader’s experience with IR [27]. In a recent study on ultralow-dose chest CT, IMR reduced objective image noise compared to FBP and iDose, but the prominent blotchy images and poor demarcation of fine structures with IMR compromised its diagnostic confidence [28]. These previous results are in good agreement with our results. In addition, the effect of high iDose levels (5–6) was similar to that of IMR application. Therefore, an intermediate level of hybrid IR (iDose between 3 and 4) may be suitable to preserve image quality while lowering the radiation dose in patients in the arms-down position.

Our study has several limitations. First, the retrospective and single-center nature of our study limited the number of patients [29]. Notably, the BMI of our study population was quite low, because it was made up of Asian patients, and most of the patients had chronic diseases and/or were critically ill. Therefore, our study results cannot be readily generalized to overweight patients. Multicenter prospective clinical studies including patients with various BMIs are required to validate our results. Second, standard-dose CT and reduced-dose CT images were scanned at different dynamic phases; therefore, the assessment of the objective and subjective image quality might be influenced by these different phases. However, repeated acquisition of CT scans in the same dynamic phase is realistically unfeasible and unethical because of increased radiation exposure to the patient. Third, objective image noise was evaluated by calculating the SD. Although several methods have attempted to evaluate image quality or noise such as grey-level co-occurrence matrices or histogram analysis, these methods were also unable to completely represent image quality. More accurate methods should be newly developed in the future, but at this moment in time, SD measurement seems to be the only widely accepted method for the quantitative analysis of CT image quality. Also, other methods used in previous studies to reduce metallic artifacts, including measuring the volume of hyperdense and hypodense artifacts, were difficult to apply in our study, because the standard-dose and reduced-dose CT scans were obtained at different dynamic phases, and beam hardening artifacts produced by the arms-down position were much weaker than those produced by metallic artifacts. Forth, subjective image quality was reviewed by two radiologists in consensus, which may decrease the generalizability of our results. Fifth, the results of this study cannot be generalized yet, because CT scanners from only one vendor were used. Further studies using IR techniques from other vendors are needed before we can generalize our study results. Finally, we did not investigate the effect of beam hardening artifacts on diagnostic efficacy, because this was a preliminary study and our main focus was investigating the feasibility of using IR at reduced-dose CT scans in this particular patient group.

In conclusion, IR algorithms can reduce beam hardening artifacts without increasing radiation dose in patients who cannot raise their arms, and an intermediate level of hybrid IR allow radiologists to obtain images of the best quality without making patients change their arm positions.

## Supporting information

S1 File Phantom study results. (DOCX)

S2 File Forms for qualitative assessment. (DOCX)

S3 File Analysis plan. (DOCX)

## References

[1] Kalra MK, Naz N, Rizzo SM, Blake MA. Computed tomography radiation dose optimization: scanning protocols and clinical applications of automatic exposure control. Curr Probl Diagn Radiol. 2005;34: 171–181. 10.1067/j.cpradiol.2005.06.002 16129235

[2] Rizzo S, Kalra M, Schmidt B, Dalal T, Suess C, Flohr T, et al Comparison of angular and combined automatic tube current modulation techniques with constant tube current CT of the abdomen and pelvis. AJR Am J Roentgenol. 2006;186: 673–679. 10.2214/AJR.04.1513 16498094

[3] McCollough CH, Bruesewitz MR, Kofler JM Jr. CT dose reduction and dose management tools: overview of available options. Radiographics. 2006;26: 503–512. 10.1148/rg.262055138 16549613

[4] Brink M, de Lange F, Oostveen LJ, Dekker HM, Kool DR, Deunk J, et al Arm raising at exposure-controlled multidetector trauma CT of thoracoabdominal region: higher image quality, lower radiation dose. Radiology. 2008;249: 661–670. 10.1148/radiol.2492080169 18936319

[5] Kahn J, Grupp U, Maurer M. How does arm positioning of polytraumatized patients in the initial computed tomography (CT) affect image quality and diagnostic accuracy? Eur J Radiol. 2014;83: e67–71. 10.1016/j.ejrad.2013.10.002 24189387

[6] Karlo C, Gnannt R, Frauenfelder T, Leschka S, Bruesch M, Wanner GA, et al Whole-body CT in polytrauma patients: effect of arm positioning on thoracic and abdominal image quality. Emerg Radiol. 2011;18: 285–293. 10.1007/s10140-011-0948-5 21472460

[7] Linsenmaier U, Krotz M, Hauser H, Rock C, Rieger J, Bohndorf K, et al Whole-body computed tomography in polytrauma: techniques and management. Eur Radiol. 2002;12: 1728–1740. 10.1007/s00330-001-1225-x 12111064

[8] Benneker LM, Bonel HM, Zumstein MA, Exadaktylos AK. A novel multiple-trauma CT-scanning protocol using patient repositioning may increase risks of iatrogenic injuries. Emerg Radiol. 2007;13: 349–351; author reply 353. 10.1007/s10140-007-0577-1 17342470

[9] Hara AK, Paden RG, Silva AC, Kujak JL, Lawder HJ, Pavlicek W. Iterative reconstruction technique for reducing body radiation dose at CT: feasibility study. AJR Am J Roentgenol. 2009;193: 764–771. 10.2214/AJR.09.2397 19696291

[10] Gervaise A, Osemont B, Lecocq S, Noel A, Micard E, Felblinger J, et al CT image quality improvement using Adaptive Iterative Dose Reduction with wide-volume acquisition on 320-detector CT. Eur Radiol. 2012;22: 295–301. 10.1007/s00330-011-2271-7 21927791

[11] Willemink MJ, Leiner T, de Jong PA, de Heer LM, Nievelstein RA, Schilham AM, et al Iterative reconstruction techniques for computed tomography part 2: initial results in dose reduction and image quality. Eur Radiol. 2013;23: 1632–1642. 10.1007/s00330-012-2764-z 23322411

[12] Park M, Chung YE, Lee HS, Choi JY, Park MS, Kim MJ, et al Intraindividual comparison of diagnostic performance in patients with hepatic metastasis of full-dose standard and half-dose iterative reconstructions with dual-source abdominal computed tomography. Invest Radiol. 2014;49: 195–200. 10.1097/RLI.0000000000000014 24300843

[13] Shin HJ, Chung YE, Lee YH, Choi JY, Park MS, Kim MJ, et al Radiation dose reduction via sinogram affirmed iterative reconstruction and automatic tube voltage modulation (CARE kV) in abdominal CT. Korean J Radiol. 2013;14: 886–893. 10.3348/kjr.2013.14.6.886 24265563 PMC3835635

[14] Yasaka K, Furuta T, Kubo T, Maeda E, Katsura M, Sato J, et al Full and hybrid iterative reconstruction to reduce artifacts in abdominal CT for patients scanned without arm elevation. Acta Radiol. 2016 2017/01/11. 10.1177/0284185116684675 284185116684675. 28068822

[15] McCollough C, Bakalyar DM, Bostani M, Brady S, Boedeker K, Boone JM, et al Use of Water Equivalent Diameter for Calculating Patient Size and Size-Specific Dose Estimates (SSDE) in CT: The Report of AAPM Task Group 220. AAPM Rep. 2014;2014: 6–23. 27546949 PMC4991550

[16] Scibelli A. iDose4 iterative reconstruction technique. Philips Healthcare Websites. http://clinical.netforum.healthcare.philips.com/global/Explore/White-Papers/CT/iDose4-iterative-reconstruction-technique. 2011.

[17] Mehta D, Thompson R, Morton T, Dhanantwari E, Shefer E. Iterative model reconstruction: simultaneously lowered computed tomotraphy radiation dose and improve image quality. Med. Phys. Int. 2013;1(2): 147–155.

[18] Lambert L, Ourednicek P, Meckova Z, Gavelli G, Straub J, Spicka I. Whole-body low-dose computed tomography in multiple myeloma staging: Superior diagnostic performance in the detection of bone lesions, vertebral compression fractures, rib fractures and extraskeletal findings compared to radiography with similar radiation exposure. Oncol Lett. 2017;13: 2490–2494. 10.3892/ol.2017.5723 28454425 PMC5403238

[19] Bayer J, Pache G, Strohm PC, Zwingmann J, Blanke P, Baumann T, et al Influence of arm positioning on radiation dose for whole body computed tomography in trauma patients. J Trauma. 2011;70: 900–905. 10.1097/TA.0b013e3181edc80e 20962679

[20] Sucha D, Willemink MJ, de Jong PA, Schilham AM, Leiner T, Symersky P, et al The impact of a new model-based iterative reconstruction algorithm on prosthetic heart valve related artifacts at reduced radiation dose MDCT. Int J Cardiovasc Imaging. 2014;30: 785–793. 10.1007/s10554-014-0379-y 24474347

[21] Habets J, Symersky P, Leiner T, de Mol BA, Mali WP, Budde RP. Artifact reduction strategies for prosthetic heart valve CT imaging. Int J Cardiovasc Imaging. 2012;28: 2099–2108. 10.1007/s10554-012-0041-5 22476910 PMC3485534

[22] Jeong S, Kim SH, Hwang EJ, Shin CI, Han JK, Choi BI. Usefulness of a metal artifact reduction algorithm for orthopedic implants in abdominal CT: phantom and clinical study results. AJR Am J Roentgenol. 2015;204: 307–317. 10.2214/AJR.14.12745 25615752

[23] Song JS, Lee JM, Sohn JY, Yoon JH, Han JK, Choi BI. Hybrid iterative reconstruction technique for liver CT scans for image noise reduction and image quality improvement: evaluation of the optimal iterative reconstruction strengths. Radiol Med. 2015;120: 259–267. 10.1007/s11547-014-0441-9 25168773

[24] Nelson RC, Feuerlein S, Boll DT. New iterative reconstruction techniques for cardiovascular computed tomography: how do they work, and what are the advantages and disadvantages? J Cardiovasc Comput Tomogr. 2011;5: 286–292. 10.1016/j.jcct.2011.07.001 21875826

[25] Yuki H, Oda S, Utsunomiya D, Funama Y, Kidoh M, Namimoto T, et al Clinical impact of model-based type iterative reconstruction with fast reconstruction time on image quality of low-dose screening chest CT. Acta Radiol. 2016;57: 295–302. 10.1177/0284185115575537 25817455

[26] Yoon JH, Lee JM, Yu MH, Baek JH, Jeon JH, Hur BY, et al Comparison of iterative model-based reconstruction versus conventional filtered back projection and hybrid iterative reconstruction techniques: lesion conspicuity and influence of body size in anthropomorphic liver phantoms. J Comput Assist Tomogr. 2014;38: 859–868. 10.1097/RCT.0000000000000145 25321625

[27] Pickhardt PJ, Lubner MG, Kim DH, Tang J, Ruma JA, del Rio AM, et al Abdominal CT with model-based iterative reconstruction (MBIR): initial results of a prospective trial comparing ultralow-dose with standard-dose imaging. AJR Am J Roentgenol. 2012;199: 1266–1274. 10.2214/AJR.12.9382 23169718 PMC3689212

[28] Laqmani A, Avanesov M, Butscheidt S, Kurfurst M, Sehner S, Schmidt-Holtz J, et al Comparison of image quality and visibility of normal and abnormal findings at submillisievert chest CT using filtered back projection, iterative model reconstruction (IMR) and iDose4. Eur J Radiol. 2016;85: 1971–1979. 10.1016/j.ejrad.2016.09.001 27776648

[29] Taylor S, Van Muylem A, Howarth N, Gevenois PA, Tack D. CT dose survey in adults: what sample size for what precision? Eur Radiol. 2017;27: 365–373. 10.1007/s00330-016-4333-3 27048530

