# Supplementary material for: Feasibility of radiation dose reduction with iterative reconstruction in abdominopelvic CT for patients with inappropriate arm positioning
Source: PLoS One. 2018 Dec 31;13(12):e0209754. doi: 10.1371/journal.pone.0209754 (PMC6312263; doi:10.1371/journal.pone.0209754)
Supplement: S1 File — (DOCX) [file pone.0209754.s001.docx]

**S1 File. Phantom study results**

A phantom study was conducted to determine the optimal degree of radiation dose reduction of CT scans without arm elevation to be similar with the dose of CT scans with arm elevation. A 256-detector row CT scanner (Brilliance iCT, Philips Healthcare, Cleveland, OH, USA) was used for this phantom study. First, an anthropomorphic phantom (Adult Male Phantom model 701-G-ATOM, CIRS, Norfolk, Virginia, USA) alone was scanned to simulate a standard-dose CT scanning with arm raising. Second, two femoral bones of a cow were located right and left of the body phantom to simulate a CT scanning without arm raising. The latter CT scan was performed with decreasing radiation dose from the standard dose using the Dose Right Index (DRI). According to this phantom study, the dose length product (DLP) increased by 22.3% when two bones were located alongside the torso (378.2 mGy·cm) compared with the case in which bones were not included in the scanning field of abdominal CT (309.0 mGy·cm). The radiation dose of reduced-dose CT scan with arm-down position using the lower DRI (80% of CT dose with arm elevation) was similar with that of standard-dose CT with arm elevation.
