# Supplementary material for: Feasibility of radiation dose reduction with iterative reconstruction in abdominopelvic CT for patients with inappropriate arm positioning
Source: PLoS One. 2018 Dec 31;13(12):e0209754. doi: 10.1371/journal.pone.0209754 (PMC6312263; doi:10.1371/journal.pone.0209754)
Supplement: S2 File — (DOCX) [file pone.0209754.s002.docx]

**S2 File. Forms for qualitative assessment**

Scoring:

1) For beam hardening artifact, image noise, artificial texture

1: much more beam hardening artifact (image noise or artificial texture) than the standard-dose CT

2: slightly more than the standard-dose CT

3: similar to the standard-dose CT

4: slightly less than the standard-dose CT

5: much less than the standard-dose CT

2) For margin sharpness and overall image quality

1: much poorer than the standard-dose CT

2: slightly poorer than the standard-dose CT

3: similar to the standard-dose CT

4: slightly better than the standard-dose CT

5: much better than the standard-dose CT

| Patient No. | Reconstruction (reduced dose) | Beam hardening  (1–5) | Artificial texture  (1–5) | Subjective image noise  (1–5) | Sharpness  (1–5) | Overall image quality  (1–5) |
| --- | --- | --- | --- | --- | --- | --- |
| 1 | FBP |  |  |  |  |  |
|  | iDose 1 |  |  |  |  |  |
|  | iDose 2 |  |  |  |  |  |
|  | iDose 3 |  |  |  |  |  |
|  | iDose 4 |  |  |  |  |  |
|  | iDose 5 |  |  |  |  |  |
|  | iDose 6 |  |  |  |  |  |
|  | IMR 1 |  |  |  |  |  |
|  | IMR 2 |  |  |  |  |  |
|  | IMR 3 |  |  |  |  |  |
| 2 | FBP |  |  |  |  |  |
|  | iDose 1 |  |  |  |  |  |
|  | iDose 2 |  |  |  |  |  |
|  | iDose 3 |  |  |  |  |  |
|  | iDose 4 |  |  |  |  |  |
|  | iDose 5 |  |  |  |  |  |
|  | iDose 6 |  |  |  |  |  |
|  | IMR 1 |  |  |  |  |  |
|  | IMR 2 |  |  |  |  |  |
|  | IMR 3 |  |  |  |  |  |
| 3 |  |  |  |  |  |  |
|  |  |  |  |  |  |  |
|  |  |  |  |  |  |  |
