# Supplementary material for: Feasibility of radiation dose reduction with iterative reconstruction in abdominopelvic CT for patients with inappropriate arm positioning
Source: PLoS One. 2018 Dec 31;13(12):e0209754. doi: 10.1371/journal.pone.0209754 (PMC6312263; doi:10.1371/journal.pone.0209754)
Supplement: S3 File — (DOCX) [file pone.0209754.s003.docx]

**S3 File. Analysis plan**

1. Comparison of quantitative image noise between standard-dose CT images and each reconstruction set of the reduced-dose CT images

- Measurement of quantitative image noise

- Standard deviation (SD) of the CT number of subcutaneous fat (including an area affected by beam hardening artifact)
- SD of the CT number of liver (including an area affected by beam hardening artifact)
- (max-min)/mean CT density

- Statistical method: paired *t*-test

2. Comparison of the qualitative scores between standard-dose CT images and each reconstruction set of the reduced-dose CT images

- Measurement of qualitative image noise using a five-point scale

- Beam hardening artifact
- Subjective image noise
- Artificial texture
- Margin sharpness
- Overall image quality

- Statistical method: Wilcoxon signed rank test
